# Long-term survival in a patient with brain metastases and leptomeningeal disease of breast cancer: a case report of a patient receiving trastuzumab-deruxtecan

**DOI:** 10.1007/s00404-025-08096-2

**Published:** 2025-07-08

**Authors:** Patricia von Kroge, Volkmar Müller, Barbara Schmalfeldt, Kerstin Riecke, Leonor Matos, Johann Kornowski, Elena Laakmann

**Affiliations:** 1 Department of Gynaecology, University Medical Center Hamburg-Eppendorf, Martinistrasse 52, 20246 Hamburg, Germany; 2 Breast Unit, Champalimaud Clinical Center, Champalimaud Foundation, Lisbon, Portugal

**Keywords:** Breast cancer, CNS metastases, Brain metastases, Trastuzumab-deruxtecan, Long-term survival, HER2-positive metastatic breast cancer, Leptomeningeal disease

## Abstract

**Introduction:**

The incidence of central nervous system (CNS) metastases in breast cancer (BC) patients is increasing, and the prognosis for those with CNS involvement, especially when accompanied by leptomeningeal disease, remains poor. We present a case of long-term survival in a patient with CNS metastases from HER2-positive BC and discuss the treatment considerations in this context.

**Case presentation:**

A 54-year-old woman with HER2-positive BC developed CNS metastases, including a highly suspicious finding for leptomeningeal disease, 2 years after initial treatment. At the time of her primary BC diagnosis, she received neoadjuvant chemotherapy, followed by breast surgery, adjuvant radiation, and anti-HER2 targeted therapy. Two years later, she developed parenchymal BM, and brain MRI revealed a leptomeningeal involvement, accompanied by neurological symptoms, including an epileptic episode. She underwent surgical resection and stereotactic radiotherapy for the parenchymal BM, followed by reinduction of trastuzumab as systemic treatment. As the disease progressed and neurological symptoms worsened, the patient received T-DM1. After further cerebral progression in 2021, therapy was switched to Trastuzumab-deruxtecan (T-DXd). Since May 2022, she has received 30 cycles of T-DXd (with a reduced dosage of 4.4 mg/kg since August 2023) without evidence of disease progression.

**Conclusion:**

Long-term survival is achievable in patients with CNS metastases from BC, even in the presence of leptomeningeal disease, especially with the use of targeted therapies like T-DXd.

## Introduction

Breast cancer (BC) is the most common type of cancer in women worldwide [1] and one of the most leading causes of death in women [2]. Due to improvement of treatment options and longer survival in metastatic BC as well as optimized brain imaging options, the incidence of central nervous system (CNS) metastases in patients with BC is increasing. The risk of developing CNS metastases in patients with metastatic BC is 30–50% [3] and may even be higher in patients with undiagnosed, asymptomatic CNS metastases [3]. The risk of developing CNS metastases also differs according to the BC subtype. Patients with HER2-positive BC develop CNS metastases in up to 40% cases [4]. Leptomeningeal metastases (LM) occur in approximately 10–12% cases at time of intracranial involvement [5]. Leptomeningeal metastasis is defined as the spread of tumor cells into the subarachnoid space and the leptomeninges [6]. Typical neurological symptoms of LM include i.e. headache, nausea or limitations in vision [6]. For the diagnosis of LM a clinical evaluation, neuroimaging such as magnetic resonance imaging (MRI) of the head and spinal cord or lumbar punction should be considered [6]. The diagnosis of LM is confirmed if tumor cells are identified in the cerebrospinal fluid. In the absence of tumor cells in liquor, the probability of the LM is high if typical MR-graphic LM signs, such as contrast agent enhancement in the meninges (especially in the region of the basal cisterns and the lumbosacral sac) and leptomeningeal signal increase in the FLAIR sequence occur and if neurological symptoms are present [6]. The diagnosis of leptomeningeal metastases can be performed if the presence of typical clinical symptoms in combination with typical MRI findings occur, as per European Association of Neuro-Oncology (EANO) -European Society of Medical Oncology (ESMO) criteria [6].

The prognosis of patients with CNS metastases is limited (especially in the presence of LM) resulting in a median overall survival (OS) for patients with LM of 3–4 months [5]. The prognosis of patients with parenchymal CNS metastases in BC without LM is considerably better with median OS differing between 4 and 25 months [3]. Regarding the median progression free survival (PFS) Darlix et al. described 5.5 months for patients with CNS metastases. The survival of patients with CNS metastases differs according to tumor biology, number of visceral metastases, previous therapies and patient age [3]. Only limited evidence is available concerning the PFS of patients with BC and LM. Currently there are different treatment options for patients with CNS metastases in BC including surgery, radiotherapy and systemic treatment such as chemotherapy and targeted therapies [7]. Surgery followed by radiotherapy is a common pathway for patients with high need of decompression in case of neurological symptoms and in cases of need for verification of tumor biology. Patchell et al. showed that the OS improved for patients having received radiotherapy after surgery vs. radiotherapy alone. Whole brain irradiation (WBRT) has no additional survival benefit over stereotactic radiotherapy but, on the contrary, implicates more side effects. So far there are several trials investigating the effect of different systematic treatment options for CNS metastases.

Current treatment options of CNS metastases such as radiotherapy and surgery rarely significantly extend survival times and are still considered as a highly palliative approach [8]. Systemic treatments are mainly used to control the local site as well as cerebral and visceral metastases [9] and have been studied mostly in the context of BMs and have rarely included patients with worse prognostic features, such as LM.

Systemic treatment options for BM differ depending on the biological subtype of the disease. For patients with newly diagnosed metastatic HER2-positive BC, the combination of trastuzumab and pertuzumab with a taxane is standard of care, independent on the presence or absence of BM. The PATRICIA trial data—published in 2021- demonstrated an ORR of 11% for patients with CNS metastases and HER2-positive BC [10]. Even though the ORR in this trial was modest, 68% of the patients treated with trastuzumab showed a clinical benefit and in two cases stable intracranial and extracranial disease for > 2 years could be demonstrated [10]. With the antibody drug conjugate T-DM1 is another treatment option for patients with advanced HER2-positive BC and CNS involvement. In studies investigating this drug, patients with asymptomatic CNS metastases were included [11].

The DESTINY-BREAST03 trial investigated the superiority of treatment with the novel antibody drug conjugate T-DXd in patients with HER2-positive metastatic BC, when compared to T-DM1 [12]. In the DESTINY-Breast03 study Cortés et al. investigated the effect of T-DXd vs. T-DM1 in patients with progressive disease after the treatment with trastuzumab plus pertuzumab and a taxane. The study showed a significant benefit for T-DXd over T-DM1 (Cortés et al. 2020). A total of 43 patients (16%) who received T-DXd and a total of 39 patients who received T-DM1 (14%) had stable CNS metastases [13]. The median OS rate at 12 month was 94.1% (95% CI 90.4–96.4) in the T-DXd group compared to 86% (95% CI 81.1–89.8) in the T-DM1 group [13]. The results of this trial showed a benefit of the PFS in all subgroups with 25.1 months when treated with T-DXd compared to 7.2 months with T-DM1 [12]. Compared to the DESTINY 03 trial, the TUXEDO-1 trial investigated the effect of T-DXd also in HER2-positive breast cancer patients with active CNS metastases.

The results of the DESTINY-Breast12, a large prospective study reporting intracranial activity of T-DXd in patients with HER2-positive metastatic BC and baseline BMs, were presented recently at the ESMO Congress 2024. This study included a total of 504 patients, 263 patients with BM at baseline (157 patients with previously treated and stable BM, and 106 with active BM) and 241 patients without BM all receiving T-DXd. The endpoints of this study was PFS and ORR. The 12-month PFS in the cohort with patients with BM at baseline was 61.6% (95% confidence interval [CI] 54.9–67.6), and 12-month CNS PFS was 58.9% (95% CI 51.9–65.3). In the non-brain metastases cohort, ORR was 62.7% (95% CI 56.5–68.8). The PFS with stable BMs at baseline was 62.9% (95% CI 54.0–70.5) and for patients with active BMs the PFS was 59.6% (95% CI 49.0–68.7) [14]. This study indicates an efficacy in of T-DXd in patients with BM (stable and active).

The HER2Climb trial investigated the treatment with tucatinib, capecitabine and trastuzumab for patients with HER2-positive BC [15]. In this trial, 612 patients with CNS metastases were included [16]. Of these, 198 patients received tucatinib, trastuzumab and capecitabine and 80 patients had stable CNS metastases, while 118 patients had active CNS metastases [16]. The control cohort also included 93 patients with CNS metastases of which 37 had stable and 57 active CNS metastases [16]. The results of this trial showed a significant benefit with an improvement in the median OS with tucatinib of 9.1 months [16]. This trial also showed a benefit for patients with active CNS metastases. The median OS was 9.6 months longer for patients with active CNS metastases receiving the combination with tucatinib compared to the patients with active CNS metastases only receiving capecitabine, trastuzumab and placebo [16].

For patients with a hormone receptor-positive BC and CNS metastases the most pronounced evidence exists for the abemaciclib-based treatment [17].

For patients with LM, radiotherapy is not recommended as a first line treatment [11]. For those patients, intrathecal therapy with methotrexate (MTX), liposomal cytarabine (Ara-C) or thioTEPA is a treatment option [11]. In general, the ESMO guideline only recommends intrathecal therapy for symptomatic patients [6].

In the majority of recently performed clinical trials, patients with active BM and LM were excluded, making available evidence of the treatment options in this context insufficient.

## Case report

In December 2015, a 54-year-old patient initially presented with HER2-positive (ER neg, PR neg, HER2 3+) invasive BC on the right side (cT2 pN0 M0). The initial therapy included neoadjuvant sequential anthracycline/taxane-containing chemotherapy in combination with trastuzumab/pertuzumab, followed by breast-conserving surgery with axillary dissection. After surgery, the patient underwent radiation of the breast including the lymphatic drainage pathways on the right. Post-neoadjuvant, the treatment with trastuzumab subcutaneous, every 3 weeks for 1 year was performed.

In August 2017, the patient presented with an epileptic seizure. In the brain MRI, a single parenchymal BM on the right frontal side was detected. A microsurgical excision was carried out followed by subsequent radiotherapy. Due to the absence of extracranial metastasis and HER2-positive BM (IHC 3+) the treatment with trastuzumab s.c. was reinduced.
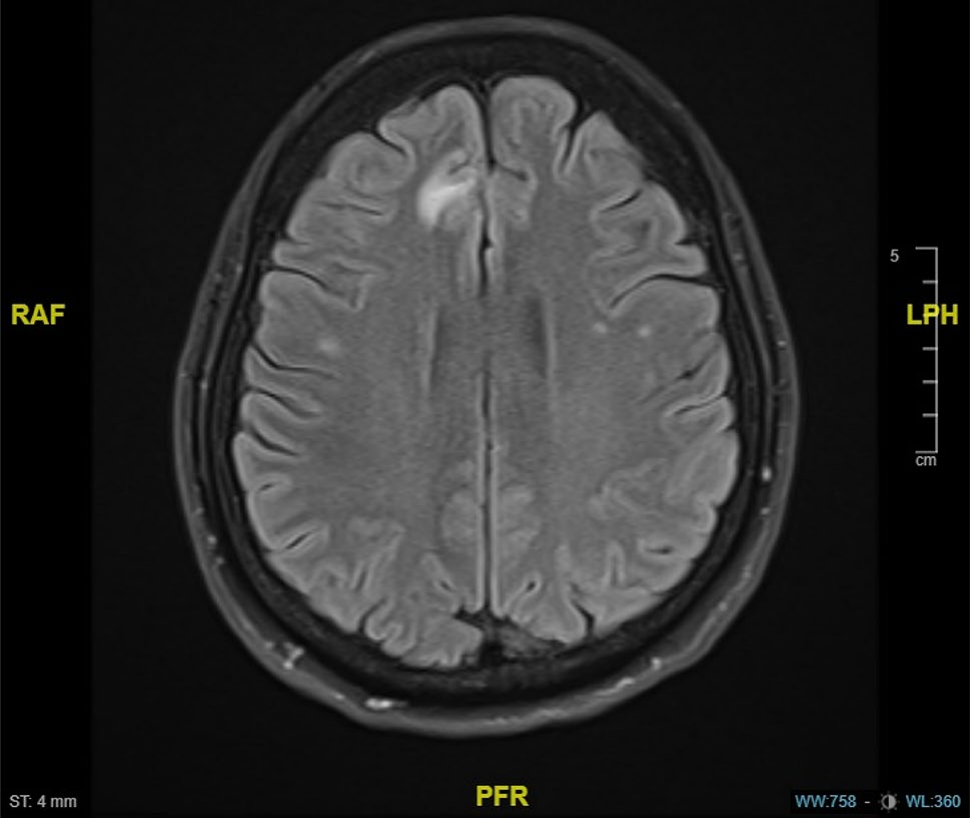


In 2018, the patient was diagnosed with bone metastases in follow-up imaging and therefore denosumab was added as antiresorptive therapy to the therapeutic regime. One year later the patient presented with progressive neurological symptoms including nausea, headaches, balance disorder and visual disturbances. The MRI showed typical signs of LM. No cytological correlate could be found in the subsequently carried out cerebrospinal fluid puncture. As the graphic signs on MRI were corresponding neurological symptoms, LM was assumed according to the EANO-ESMO guideline definition [6]. In further course of disease MR-graphic signs of LM progression were detected. Further liquor punctions were rejected by the patient. Subsequently a radiation of the neuroaxis was performed. While the visceral disease remained stable, therapy with trastuzumab was continued.

In January 2021, the systemic treatment was switched to T-DM1 due to a further cerebral progression and visceral stable disease. In this MRI the patient had a progressive disease in the LM infestation.
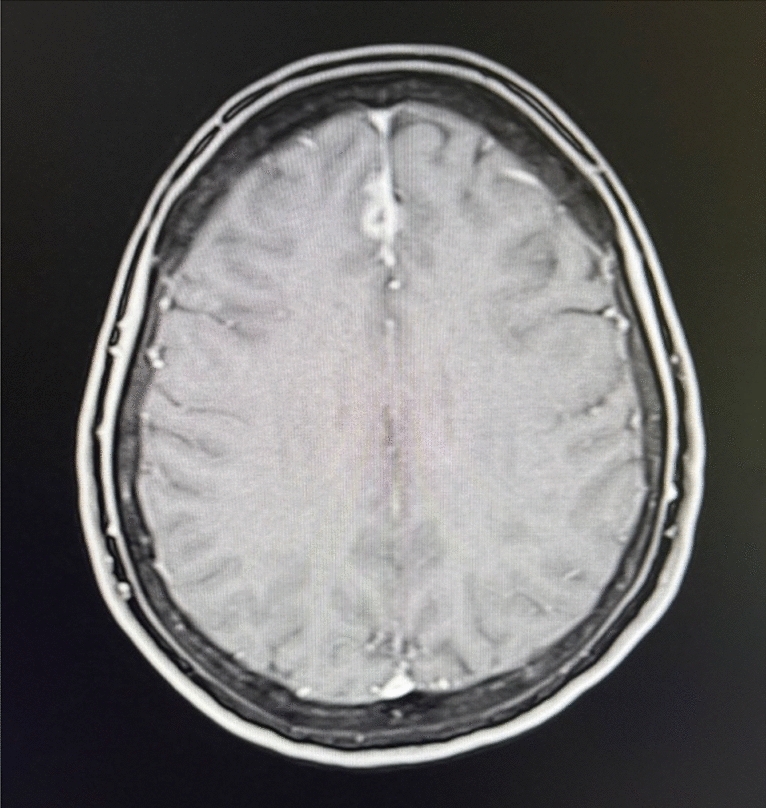


The patient received T-DM1 until the next MR-graphic cerebral progression in May 2022. 
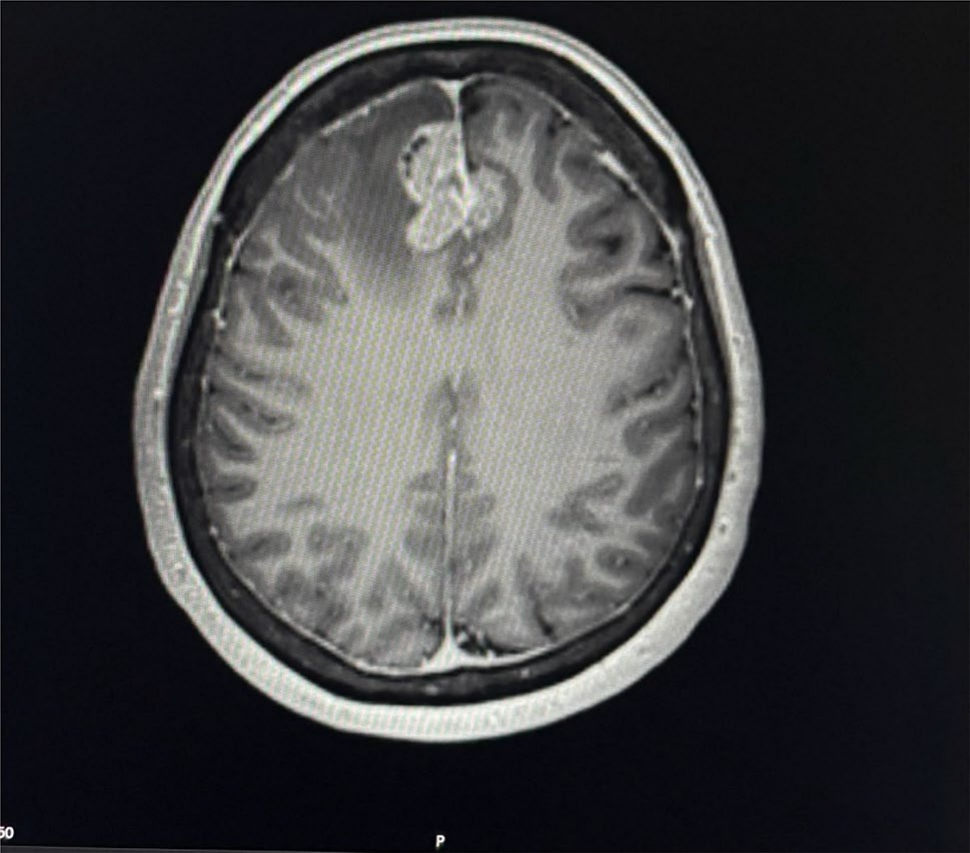


The treatment was then alternated to T-DXd 5.4 mg/kg every 3 weeks. The patient initially tolerated the therapy very well. After a few cycles of therapy, the patient experienced a significant improvement of the neurological symptoms like headache, dizziness and nausea. Follow-up imaging showed both visceral and cerebral stable disease ever since and therefore T-DXd application was continued. In August 2023, the initial dose of 5.4 mg/kg every 3 weeks was reduced to 4.4 mg/kg every 3 weeks due to elevated liver parameters and recurrent neutropenia. Due to continued response to therapy and the patient’s wish, therapy with 4.4 mg/kg has now been extended to every 4 weeks for 4 months. The patient has already received 30 cycles of T-DXd without any signs of progression. Until now the patient has no recurrence of neurological complaints.

In total, the patient survived since 7 years after the diagnosis of CNS metastases and suspected LM.
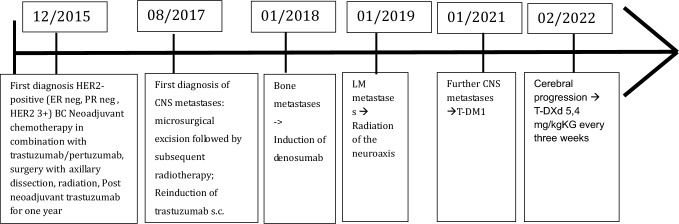


## Discussion

As the treatment options for (advanced) BC continued to develop and improve, patients’ survival is extending and thus, the incidence of CNS metastases has increased [9]. The development of CNS metastases continues to be a drastic change for patients with BC with profound effects on their quality of life as well as their survival [1]. The arising of CNS metastases is predominately seen in patients with BC that is biologically HER2-positive or triple-negative [1].

The evidence of the efficacy of systemic treatment options in patients with active CNS metastases with LM in HER2-positive BC is insufficient. Patients with a leptomeningeal disease are often excluded from the clinical trials and thus therapeutic options for this population represents a highly unmet need.

We have described in this clinical case, a patient with a long-term survival after the diagnosis of CNS metastases and suspicion of LM, with the use of HER2-targeted treatment, including the novel antibody drug conjugate T-DXd, with a consistent and maintained response to this treatment, with tolerable toxicity and thus preserved quality of life.

According to the analysis of Klar et al., about 20–30% of patients BC achieve long-term-survival (defined as survival ≥ 5 years) [18]. Our patient shows a long-term survival over 7 years and continues to have stable disease and a very good quality of life. Long-term survivors in cases of metastatic BC in general are typically younger at the diagnosis of metastatic disease, are premenopausal and the most common biological subtype is estrogen receptor -positive, progesterone receptor-positive, and HER2-positive [18]. When considering these factors our patient was 56-years at the diagnose of CNS metastases, had a HER2-positive BC and therefore showed the commonly predictive factors for long-term survival.

T-DXd has demonstrated durable antitumor activity in previously treated patients with HER2-positive metastatic BC. The efficacy in patients with active BM has been investigated in several trials: Destiny Breast 12, DEBBRAH, TUXEDO, ROSET. The efficacy and safety of T-DXd in patients with LM was until now analyzed in a limited amount of studies with small sample size. In the DEBBRAH study, 7 patients with LM of HER2-positive BC were treated with T-DXd. The results of this trial showed a median OS of 13.3 months for patients with LM receiving T-DXd. The median PFS was 8.9 month. These results are very promising and suggest a further investigation of T-DXd and HER-2 positive BC with LM [19]. The results of the DEBBRAH trial correspond well to the observation in our case report.

Another study worth mentioning is the ROSET-BM trial published in 2023 [20]. This trial also included 113 patients with HER2-positive BC with CNS metastases who were treated with T-DXd. 73 patients had active BM without LM and 17 patients had active BM with LM. The median PFS in the total population was 16.1 month and the 12-month OS was 74.9%. In the sub analysis for the patients with LM the 12-month PFS was 60.7% (95% CI 34.5–79.1) and the OS rates 87.1% (95% CI 57.3–96.6%). These results also indicate a durable treatment response under T-DXd for patients with LM.

The aim of this presentation was to report a case of extremely long survival and good quality of life in a patient with HER2-positive metastatic BC and active CNS metastases with suspicion of LM including a durable treatment response under T-DXd.

## Conclusion

We report a case of a patient with long-term survival with active CNS metastases of a metastatic HER2-positive BC and suspicion of LM. Under the treatment with T-DXd a survival of 25 months could be achieved without a recurrence of any neurological symptoms. Even though the current evidence of efficacy of T-DXd in patients with LM is still very limited, the current data also suggests an effect of T-DXd in this cohort of patients. Further evidence from prospective clinical trials is needed to confirm the effectiveness of T-DXd in patients with HER2-positive BC and LM.

## Data Availability

No datasets were generated or analysed during the current study.
